# Fecal microbiota transplantation from a healthy pouch donor for chronic pouchitis: a proof-of-concept study

**DOI:** 10.1080/19490976.2025.2510464

**Published:** 2025-05-25

**Authors:** Sabrina Just Kousgaard, Sebastian Mølvang Dall, Mads Albertsen, Hans Linde Nielsen, Ole Thorlacius-Ussing

**Affiliations:** a Department of Gastrointestinal Surgery, Aalborg University Hospital, Aalborg, Denmark; b Department of Clinical Medicine, Aalborg University, Aalborg, Denmark; c Center for Microbial Communities, Aalborg University, Aalborg, Denmark; d Department of Clinical Microbiology, Aalborg University Hospital, Aalborg, Denmark

**Keywords:** Pouchitis, IPAA, fecal microbiota transplantation, microbiome, metagenomics

## Abstract

Chronic pouchitis is a common complication after ileal pouch-anal anastomosis (IPAA) with limited treatment options. In this case series, we aimed to investigate clinical and microbiome changes, as well as adverse events, associated with using fecal microbiota transplantation (FMT) from a donor with a normal functioning IPAA to induce remission in patients with chronic pouchitis. Methods The study was a case-series including a 4-week intervention period and 12-month follow-up. Patients with chronic pouchitis who met the inclusion criteria were recruited from the Department of Gastrointestinal Surgery at Aalborg University Hospital, Denmark. Participants received FMT derived from a donor with a normal functioning IPAA. Treatment was administered by enema daily for two weeks, then every other day for two more weeks. Disease severity and quality of life (QoL) were accessed at baseline and 30-day follow-up. Clinical remission was defined as Pouchitis Disease Activity Index (PDAI) <7. Fecal samples from participants, healthy donors, and the IPAA donor were analyzed using shotgun metagenomic sequencing. Results Three patients with chronic pouchitis were included and completed the treatment protocol and follow-up visits. At the 30-day follow-up, all participants achieved clinical remission with reduced endoscopic inflammation. The median total PDAI score decreased from 8 (range 10–8) at baseline to 6 (range 6–5) at 30 days. Two participants reported improved QoL, while one reported no change. Few mild, self-limited adverse events were reported by all participants during treatment, with no serious events. Principal component analysis of fecal samples distinguished two clusters: healthy donors and the IPAA donor, with participant samples forming a separate cluster Conclusion We observed that all participants achieved clinical remission with reduced endoscopic inflammation following a 4-week FMT intervention. Adverse events were mild and self-limited. Metagenomic analysis revealed distinct microbiome clusters between IPAA donor and recipients, both of which differed from those of healthy donors.

## Introduction

Pouchitis is the most common long-term complication following ileal pouch-anal anastomosis (IPAA) surgery, with reported incidence rates as high as 59% in Western countries.^1,2^ Treatment with ciprofloxacin and/or metronidazole is the standard first-line therapy; however, this approach often proves ineffective for chronic pouchitis.^3^ Chronic pouchitis occurs in up to 20% of patients within five years of surgery and poses significant treatment challenges.^4,5^ Biologic therapies such as infliximab, adalimumab, vedolizumab, and ustekinumab have been investigated for the treatment of chronic antibiotic-dependent or antibiotic-refractory pouchitis, demonstrating response rates ranging from 28% to 51%.^6^ Furthermore, treatment with small molecule therapies, such as Janus kinase inhibitors, may play a role in the management of chronic pouchitis by decreasing the pro-inflammatory load.^7^ In severe cases, it may necessitate surgical removal of the IPAA due to pouch failure.^8^

The pathogenesis of pouchitis remains incompletely understood, with the gut microbiota hypothesized to play a contributory role. Alterations in gut microbiota has been associated with ileal pouch-anal anastomosis (IPAA) patients experiencing pouchitis, in contrast to those without inflammation.^9,10^

Fecal microbiota transplantation (FMT) has been investigated as a treatment for chronic pouchitis in several studies, however randomized placebo-controlled trials have not demonstrated a benefit of FMT over placebo.^11^ To date, all studies have used healthy fecal donors with an intact colon.

In this case series, we aimed to investigate clinical and microbiome changes, as well as adverse events, associated with using FMT from a donor with a normal functioning IPAA to induce remission in patients with chronic pouchitis.

## Methods

### Study design, setting, and patients

The case-series proof-of-concept study was conducted at the Department of Gastrointestinal Surgery, Aalborg University Hospital, Aalborg, Denmark, from March 2021 to February 2022. The study used FMT from a donor with a normally functioning IPAA to treat three patients with chronic pouchitis.

### Inclusion and exclusion criteria for participants

Eligible patients were enrolled from the same department. To qualify, participants needed a total Pouchitis Disease Activity Index (PDAI) score of at least 7, with a clinical symptom sub-score of at least 3 and both endoscopic and histologic sub-scores of at least 1.^12^ Chronic pouchitis was defined as having three or more episodes of pouchitis within the past year and/or experiencing persistent symptoms for more than four weeks despite antibiotic therapy. Both patients with antibiotic-dependent and antibiotic-refractory chronic pouchitis were included.

Patients were excluded if they had secondary pouchitis (e.g., Crohn’s disease), immunosuppression (such as human immunodeficiency virus, prolonged corticosteroid use for over three months, prior anti-TNF-alpha therapy, or chemotherapy), recent probiotic use within the past month, a history of anaphylaxis, or significant food allergies. Additional exclusion criteria included pregnancy, planned pregnancy, or breastfeeding. Finally, patients were excluded if their stool test was positive for enteric bacterial pathogens, including *Salmonella*, *Campylobacter*, *Yersinia*, *Shigella*, *Vibrio*, toxin-producing *Clostridioides difficile*, and diarrheagenic *Escherichia coli* (including Shiga toxin-producing *E. coli* (STEC)).

### Donor selection and stool processing

The donor had a normal functioning IPAA and was recruited from the Department of Gastrointestinal Surgery.

The donor had no recorded episodes of pouchitis, no symptoms of pouch dysfunction, and no history of antibiotic use for pouchitis.

Donor screening followed international guidelines for FMT,^13^ including a health questionnaire and blood and fecal tests; see the supplemental material for donor selection and stool processing procedures.

### Treatment design

The treatment regimen involved daily FMT administration via enema with 100 ml suspended fecal material for 14 consecutive days, followed by every other day for the subsequent 14 days. Prior to FMT, participants ceased any antibiotic treatment at least one day in advance. The initial FMT dose was supervised at the outpatient clinic, while subsequent doses were self-administered at home with detailed instructions.

At baseline (before intervention) and at the primary 30-day follow-up (after intervention), patients underwent pouchoscopy for biopsy collection, as well as fecal and blood sampling. Fecal samples were also collected during treatment at days 5, 10, 16, and 28. The complete Pouchitis Disease Activity Index (PDAI) and Quality of Life (QoL) assessment using the Short Inflammatory Bowel Disease Questionnaire (SIBDQ)^14^ was conducted.

Fecal samples, symptom scores, and stool frequency were collected at 1, 3, 6, and 12 months, with the follow-up period concluding at 12 months.

Adverse events (AEs) were documented throughout the study period.

### Microbiome sequencing

DNA was extracted from the fecal samples with the DNeasy® 96 Powersoil® Pro QIAcube HT kit as described by Jensen et al.^15^ Metagenomic sequencing was performed on the NovaSeq Illumina platform. To compare metagenomic sequencing results, we used fecal samples from healthy donors with an intact colon, included in the MicroPouch study.^16^ The microbiome sequencing protocol is described in the supplemental material.

### Statistical analysis

Baseline demographics are presented in a descriptive case-series table. Effectiveness for clinical outcomes is presented in a table with median and range for PDAI score including sub-scores, stool frequency, fecal calprotectin and SIBDQ. Clinical remission was defined as a total PDAI < 7 points. Adverse events are presented in a table using frequencies and percentages.

Microbiome data analysis was conducted in R and RStudio using the packages tidyverse,^17^ vegan,^18^ ggplot2,^19^ ggpubr,^20^ and ampvis2.^21^ Microbial richness was defined as the number of species with a relative abundance greater than zero, while alpha diversity was measured using the Shannon index. Similarity to the IPAA donor was assessed using the Sørensen coefficient for relative abundances and Bray-Curtis similarity for Hellinger-transformed abundances. Beta diversity was further explored through Principal Component Analysis (PCA) based on Hellinger-transformed relative abundances.

Data was analyzed using R v4.1.0 and STATA® V.17.0 (StataCorp LP, Texas, USA). A P-value <0.05 was considered statistically significant.

### Ethics

The study was performed adhering to the requirements of Good Clinical Practice and the Revised Declaration of Helsinki. All patients included provided signed written informed consent to participate. Consent for participation could be withdrawn at any time during the study period. The Regional Research Ethics Committee of Northern Jutland, Denmark, approved the study (project number *N*-20150021). The study was registered at ClinicalTrials.gov (trial number NCT04820413).

## Results

### Case-series population

Three patients with chronic pouchitis were included and completed the 28-day FMT protocol using a donor with a normally functioning IPAA. All patients attended follow-up visits through the 12-month study period. Baseline characteristics are summarized in Table 1.

**Table 1.** Baseline characteristics of the case-series study population (n = 3).

Characteristic	Patient 1	Patient 2	Patient 3
Gender	Female	Female	Female
Age, years	40	66	57
Ethnicity	Caucasian	Caucasian	Caucasian
BMI, kg/m^2^	19.8	18.9	25.4
Years since IPAA surgery	8	29	24
PDAI	8	10	8
Stool frequency	20	8	13
CRP, mg/L	0.6	0.6	1.8
Leucocytes, x10^9^/L	4.1	8.6	4.8
Fecal calprotectin, mg/kg	37	512	393
Types of chronic pouchitis	Antibiotic refractory	Antibiotic dependent	Antibiotic refractory
Number of courses of antibiotics the year up to inclusion	3	2	2
Medication	Codeine	Imodium, Alendronate	Vagifem
Participants with any other disease besides pouchitis	–	Osteoporosis	–

Abbreviations: BMI, body mass index; CRP, C-reactive protein; IPAA, ileal pouch-anal anastomosis; PDAI, pouchitis disease activity index.

### Clinical outcomes

At the 30-day follow-up, all participants achieved clinical remission with reduced endoscopic inflammation. In addition, one of the three participants achieved clinical remission along with a ≥ 3 points reduction in the PDAI from baseline at the 30-day follow-up after FMT.

The median total PDAI score decreased from a baseline median of 8 (range 10–8) to a median of 6 (range 6–5) at the 30-day follow-up. The PDAI sub-scores, clinical PDAI (cPDAI) and endoscopic PDAI (ePDAI), decreased following FMT at the 30-day follow-up, while the histologic PDAI (hPDAI) score remained unchanged (see Table 2).

**Table 2.** Table of efficacy for clinical outcomes and safety assessment in the study population (n = 3).

**Outcome**	**Fecal microbiota transplantation (n = 3)**			
Participants in clinical remission, *n (%)*	3 (100%)			
Participants in clinical remission and PDAI ≥ 3 point reduction, *n (%)*	1 (33%)			
**Clinical outcomes from baseline to 30-day follow-up**				
	**Inclusion**		**30-day follow-up**	
PDAI, *median (range)*	8 (10–8)		6 (6–5)	
Stool frequency, *median (range)*	13 (20–8)		10 (18–8)	
Fecal calprotectin, mg/kg, *median (range)*	393 (512–37)		430 (541–25)	
cPDAI, *median (range)*	4 (4–4)		2 (3–2)	
ePDAI, *median (range)*	3 (5–3)		2 (3–1)	
hPDAI, *median (range)*	1 (1–1)		1 (2–0)	
SIBDQ, *median (range)*	32 (44–22)		36 (46–22)	
**Clinical outcomes at 1-, 3-, 6- and 12-months follow-up**				
	**1 month**	**3 months**	**6 months**	**12 months**
Stool frequency, *median (range)*	12 (18–7)	12 (18–7)	12 (20–7)	18 (18–10)
cPDAI, *median (range)*	3 (4–1)	3 (3–1)	2 (4–1)	2 (3–2)
**Adverse events**				
Total participants with AE, *n (%)*	3 (100%)			
Total participants with AE reported during treatment, *n (%)*	3 (100%)			
Total participants with AE reported at follow-ups, *n (%)*	0 (0%)			
Total SAE, *n (%)*	0 (0%)			
Death, *n (%)*	0 (0%)			
Withdrawal owing to any AE, *n (%)*	0 (0%)			
Withdrawal owing to any SAE, *n (%)*	0 (0%)			
Total AEs, *n*	8			
AEs of special interest				
Abdominal pain, *n*	3			
Discomfort, *n*	1			
Other reported AEs				
Itching, *n*	2			
Bloated, *n*	1			
Dizziness, *n*	1			

Abbreviations: AE, adverse event; cPDAI, clinical pouchitis disease activity index; ePDAI, endoscopic pouchitis disease activity index; hPDAI, histologic pouchitis disease activity index; PDAI, pouchitis disease activity index; SAE, serious adverse event; SIBDQ, Short Inflammatory Bowel Disease Questionnaire.

Stool frequency and cPDAI score decreased after FMT, which was retained for clinical PDAI throughout follow-ups. The improvement in stool frequency was maintained until the 3-month follow-up, at which point it increased (see Table 2).

### Quality of life

Two participants reported an improvement in QoL, as indicated by an increase in their SIBDQ scores following FMT. This improvement was observed at the 30-day follow-up compared to their baseline scores. In contrast, one participant did not experience any change in their SIBDQ score after FMT (see Table 2).

### Adverse events

All participants reported one or more AEs; with a range of 2–3 AEs per participant, primarily abdominal pain (see Table 2). AEs occurred exclusively during the treatment period and no serious AEs were reported.

### Microbiome analysis

All donor samples were successfully sequenced and passed quality control. One patient sample from patient 2 at 12-month follow-up was excluded from the analysis due to insufficient sequencing depth.

**Figure 1.**
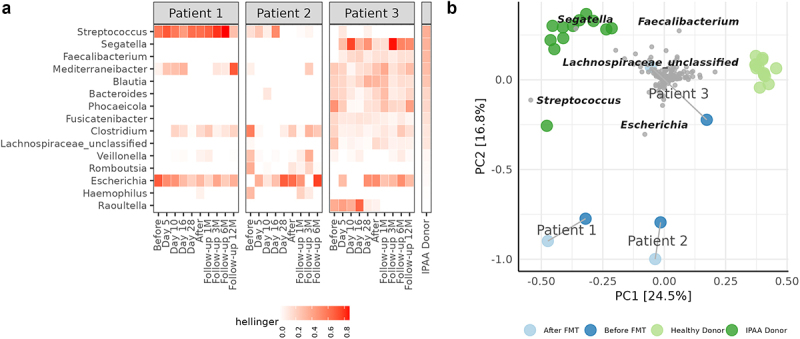
(a) Microbial composition of fecal samples. The top 15 most abundant genera based on the weighted mean of Hellinger transformed relative abundance for samples before treatment and donor (weighted by the inverse number of samples in each group). The genera are ordered according to the mean Hellinger transformed relative abundance in donor fecal samples. (b) Principal component analysis based on Hellinger transformed relative abundance of healthy donor samples (light green color), normally functioning IPAA donor (dark green color), and patient samples before (dark blue color) and after (light blue color) treatment. Grey dots are species. The genus of the 5 most extreme species is named. Grey line connects patient samples before and after treatment at 30-day follow-up.

**Figure 2.**
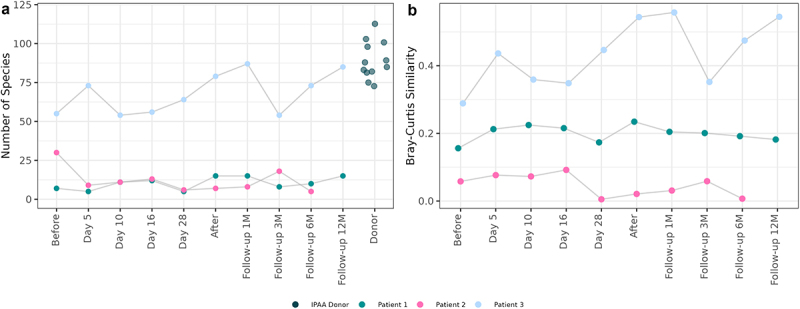
(a) Microbial alpha diversity of patients and donor fecal samples measured by the number of species. (b) Median Bray-Curtis similarity of patient fecal microbiome to donor microbiomes.

At inclusion, patient 1 and 2 exhibited similar gut microbiomes, dominated by the two genera *Escherichia* and *Streptococcus*, in contrast to patient 3, whose more diverse microbiome including several genera, resembling that of the IPAA donor (Figure 1(a)). Throughout the FMT treatment and follow-up, the fecal microbiome of patients 1 and 2 had a lower alpha diversity and richness compared to the donor microbiome (Figure 2(a)). Patient 3 had higher species richness comparable to the donor when starting the treatment, which decreased during treatment. For patient 1, microbiome richness and diversity increased during treatment but decreased to baseline levels at the 3-month follow-up. Notably, at baseline the microbiome of patient 1 had a ratio of *Escherichia* to *Streptococcus* of 2:1, which inverted following treatment. Beta-diversity increased for patient 1 during treatment until day 28, whereafter similarity to the donor microbiome dropped to the level at baseline (Figure 2(b)). For patient 2 similarity to the donor decreased during treatment (Figure 2(b)). For patient 3, an increase in similarity to the donor was observed before and after treatment, however a sharp decrease was observed at the 3-month follow-up (Figure 2(b)).

Principal component analysis (PCA) of samples from the IPAA donor and 13 healthy fecal donors with an intact colon in the MicroPouch study^16^ revealed two distinct clusters separating healthy donors and the IPAA donor (Figure 1(b)). Samples from the participants were separated from the two donor clusters, however treatment for patient 3 resulted in a microbiome shift toward the IPAA donor cluster (left-to-right).

## Discussion

The key findings of this case-series were that all participants achieved clinical remission and showed reduced endoscopic inflammation at the 30-day follow-up. FMT using a donor with a normally functioning IPAA appeared safe, with only mild, self-limiting AEs reported. Metagenomic analysis revealed distinct clustering between healthy donors, the IPAA donor and the patient microbiomes.

Previous clinical trials have investigated FMT for chronic pouchitis, all using stool from healthy donors with an intact colon. Three randomized placebo-controlled trials have been published. Herfarth et al.^22^ found no efficacy of FMT compared to placebo using a single endoscopic dose followed by daily oral treatment for two weeks. The study was prematurely terminated after enrolling six patients due to a low clinical remission rate and poor FMT engraftment. The second study, conducted by Karjalainen et al.^23^ with 26 participants, found no difference in relapse-free survival between donor FMT and autologous FMT. Patients received two treatments at weeks 0 and 4 via endoscopy and transanal catheter. The third study by Kousgaard et al.^16^ with 30 participants reported no significant difference in clinical remission rates between the FMT and placebo groups. Additionally, patients treated with FMT experienced significantly more AEs.

To our knowledge, this proof-of-concept study is the first to investigate the use of FMT from a donor with a normally functioning IPAA in patients with chronic pouchitis. The treatment was considered safe, with only few AEs reported, and fewer AEs than reported in a previous trial using a donor with an intact colon (eight reported AEs in three participants vs 86 reported AEs in 12 participants).^16^ All treated participants achieved clinical remission with reduced endoscopic inflammation at the 30-day follow-up. However, the improvement in clinical symptoms was not well-maintained at long-term follow-up. To sustain long-term therapeutic effects, maintenance treatment with repeated FMT or an optimized dosing regimen may be necessary to prolong clinical efficacy. Determining the optimal frequency and delivery method of FMT could play a crucial role in achieving durable responses and preventing relapse in patients.

The hypothesis of using a patient with a normally functioning IPAA as a fecal donor originated from the idea that the environment in an IPAA differs significantly from that of a healthy colon. It is well-established that after IPAA surgery, the ileum used to construct the pouch undergoes morphological changes, such as the loss or blunting of villi and the development of crypts, resulting in a more colon-like structure.^24,25^ Additionally, the gut microbiota in the IPAA undergoes significant changes after stoma closure, evolving to resemble the predominant bacterial species found in normal colonic microbiota.^26^ However, there are notable differences between the microbiota composition of an IPAA and a healthy colon, with the IPAA microbiota exhibiting reduced microbial diversity and richness.^27^ The metagenomics analysis demonstrated clear and distinct clusters when comparing the microbiomes of donors and patients, underscoring significant compositional differences in their microbial communities. This finding highlights the potential therapeutic utility of using the microbiome of a donor with a normally functioning IPAA to treat patients suffering from chronic pouchitis. However, the limited microbial convergence observed between recipients and the donor – with only one patient clustering closely with the donor post-FMT – highlights the potential impact of individual variability on FMT responsiveness. This case-series has several limitations. Only three patients with chronic pouchitis were included in this preliminary proof-of-concept study, making the results hypothesis-generating, and warranting cautious interpretation. Furthermore, in the absence of a placebo or standard-care comparator, it is not possible to establish a definitive causal relationship between FMT using a normally functioning IPAA donor and clinical improvement. As a result, the observed benefits may be influenced by placebo effects or natural disease fluctuations and should therefore be interpreted with caution. Due to the small sample size, the metagenomic analysis of FMT effects was inconclusive. The baseline microbiome composition of the participants varied considerably, with some exhibiting a highly disrupted microbial profile. Additionally, there was no prespecified antibiotic washout period or antibiotic pre-treatment prior to study enrollment, which could bias the initial microbiome assessment. However, all participants had a long history of antibiotic treatments. Lastly, the short duration between the completion of FMT treatment and follow-up may have influenced the microbiome assessment.

Further research investigating the microbiome and genetic differences between patients with chronic pouchitis and those with a normally functioning IPAA is needed before proceeding with larger, placebo-controlled studies on the use of FMT from normally functioning IPAA donors for the treatment of chronic pouchitis. This research could provide valuable insights into the underlying factors that influence FMT outcomes, such as host immune responses, microbial engraftment, and genetic predispositions, thereby improving the design and targeting of future clinical trials.

## Conclusion

In a case series with three participants with chronic pouchitis, we observed that all patients achieved clinical remission with reduced endoscopic inflammation following a 4-week FMT intervention. Adverse events were mild and self-limited. Metagenomic analysis revealed distinct microbiome clusters between donor and recipients. However, this was a preliminary proof-of-concept study and further research involving larger cohorts including placebo or standard-care comparator is needed to confirm the potential benefits of FMT from a normally functioning IPAA donors for treating chronic pouchitis.

## Supplementary Material

Supplemental text 100225.docx

## Data Availability

The authors confirm that the data supporting the findings of this study including individual participant data and study material relevant to the study are available within the article or uploaded as supplementary material. The microbiome data that support the findings of this study are openly available in NIH Sequencing Read Archive at https://www.ncbi.nlm.nih.gov/sra/., reference number PRJEB80556 and PRJEB66493. The raw patient data that support the findings of this study are available on request from the corresponding author, SJK. The raw patient data is not publicly available because it contains patient information that could compromise the privacy of research participants. The code for reproducing figures are available at: https://github.com/SebastianDall/MicroPouch_NP.

## Abbreviations

AEs: Adverse events
cPDAI: clinical pouchitis disease activity index
ePDAI: endoscopic pouchitis disease activity index
FMT: Fecal microbiota transplantation
hPDAI: histologic pouchitis disease activity index
IPAA: Ileal pouch-anal anastomosis
PCA: Principal component analysis
PDAI: Pouchitis Disease Activity Index
QoL: Quality of Life
SIBDQ: Short Inflammatory Bowel Disease Questionnaire
