# Supplementary material for: Fecal microbiota transplantation from a healthy pouch donor for chronic pouchitis: a proof-of-concept study
Source: Gut Microbes. 2025 May 25;17(1):2510464. doi: 10.1080/19490976.2025.2510464 (PMC12118383; doi:10.1080/19490976.2025.2510464)
Supplement: Supplemental text 100225.docx [file KGMI_A_2510464_SM8077.docx]

**Supplemental text**

*Donor Selection and Stool Processing*

*Donor screening protocol*

Screening of the recruited individual was conducted following the international guidelines for FMT, assessed using a questionnaire, and blood and fecal tests before and after fecal donations for a period of one month.^1^

Fecal donor screening questionnaire inclusion criteria:

- 20-65 years of age.
- Previously and currently healthy (except for prior Ulcerative Colitis and ileal pouch-anal anastomosis surgery).
- Body mass index (BMI) between 18.5 and 24.9 kg/m^2^.
- No consumption of pharmaceutical or natural medicines.

Fecal donor screening questionnaire exclusion criteria:

- Known chronic inflammatory disease including Crohn’s disease, coeliac disease, rheumatoid arthritis or another autoimmune joint disease, disseminated sclerosis, psoriasis.
- Diabetes mellitus.
- Acute bowel infection with diarrhea and/or rectal bleeding within the previous six months.
- Antimicrobial therapy within the previous six months.
- Tattoo or body piercing within the previous six months.
- Participation in high-risk sexual behaviors defined as a high risk of being infected with sexually transmitted infection.
- Travel to area with a high occurrence of infectious diseases within the previous six months.
- Blood transfusion within the previous six months.

Fecal and blood screening exclusion criteria:

- Positive stool sample for:
  - Intestinal pathogenic bacteria (*Clostridioides difficile* toxin, *Campylobacter*, *Salmonella*, *Shigella*, *Yersinia*, diarrhea-genic *Escherichia coli*).
  - Intestinal pathogenic viruses (Adenovirus, Rotavirus, Norovirus, Enterovirus, Parechovirus).
  - Intestinal pathogenic parasites (*Entamoeba histolytica, Cryptosporidium parvum/hominis, Giardia lamblia,* worms).
  - Multi-resistant bacteria (Vancomycin-resistant *Enterococcus*, Carbapenemase- and Extended-spectrum beta-lactamases-producing Organisms)
  - *Helicobacter pylori*.
  - SARS-CoV-2 (during the COVID-19 pandemic).
- Positive blood screening for human immunodeficiency virus, hepatitis A virus, hepatitis B virus, hepatitis C virus, cytomegalovirus, Epstein-Barr virus, or Syphilis.
- Fecal calprotectin >50 µg/g.

*Stool processing protocol*

The fecal donor was equipped with a cooling box and frozen freezer packs. Immediately after producing the fecal sample for donation, the fecal donor was instructed to place the container with the sample in the cooling box surrounded by frozen freezer packs. The fecal donor delivered the sample with a delivery note to the Department of Gastrointestinal Surgery, Aalborg University Hospital, Aalborg within a maximum of 3 hours after defecation. After receiving the sample, it was processed in the laboratory at the Department of Clinical Microbiology, Aalborg University Hospital, Aalborg, Denmark. Each fecal sample was divided into portions of 50 grams. For each portion, 100 ml of sterile water was added, and the feces were homogenized manually using a blender (Braun MQ 325). The homogenized sample was filtered using a Seward Stomacher® filtration Bag. After filtration, the sample was mixed with glycerol (100% dissolution) to a final concentration of 10% glycerol to ensure freeze protection. The final mixture was stored in a sterile enema bottle of 100 ml mixture per bottle at -80^o^C. The final amount of feces in the enema bottle was not estimated. This was stored latest four hours after delivery. Anaerobic preparation of the donated stool was not used. The enema bottles were stored prior to use at -80^o^C for a maximum one year, after which they were destroyed.

*Microbiome sequencing*

*DNA extraction, Library Preparation, and Sequencing*

DNA was extracted with the DNeasy® 96 Powersoil® Pro QIAcube HT using a slightly modified protocol as described by Jensen et al.^2^

Metagenome libraries were prepared with Illumina DNA prep using a downscaled reagent volume protocol (1:10) optimized for i-DOT one capable of handling liquids in the nanolitre-scale.^2^ Sequencing was performed on the NovaSeq Illumina platform. Samples were sequenced to a depth of a median of 4.5 Gb.

*Sequence preprocessing and Taxonomic Profiling*

Metagenomic sequences were adapter trimmed, quality filtered, and deduplicated with fastp^3^ (parameters: --correction, --detect_adapter_for_pe, --cut_right, --cut_right_window_size 4, --cut_right_mean_quality 20, average_qual 25, --length_required 100, --dedup, --dup_calc_accuracy 6). Metagenomic sequences that passed quality control were mapped to the human genome, hg38, using bowtie2 for host DNA removal.^4^ Each sample were subsequently subsampled without replacement to 2M paired-end reads. Samples containing less than 2M paired-end reads were discarded from the analysis.

Lastly, a microbial community profile was generated for metagenomic samples passing quality control with MetaPhlAn4 using the mpa_vJun23_CHOCOPhlAnSGB_202307 marker gene database.^5^

**References**

1. Cammarota G, Ianiro G, Kelly CR, et al. International consensus conference on stool banking for faecal microbiota transplantation in clinical practice. Gut 2019;68:2111–2121.

2. Jensen TBN, Dall SM, Knutsson S, et al. High-throughput DNA extraction and cost-effective miniaturized metagenome and amplicon library preparation of soil samples for DNA sequencing. PLoS One 2024;19:1–16.

3. Chen S, Zhou Y, Chen Y, et al. fastp: an ultra-fast all-in-one FASTQ preprocessor. Bioinformatics 2018;34:i884–i890.

4. Langmead B, Salzberg SL. Fast gapped-read alignment with Bowtie 2. Nat Methods 2012 94 2012;9:357–359.

5. Blanco-Míguez A, Beghini F, Cumbo F, et al. Extending and improving metagenomic taxonomic profiling with uncharacterized species using MetaPhlAn 4. Nat Biotechnol 2023;41:1633–1644.
